# Biomonitoring of Exposure in Farmworker Studies

**DOI:** 10.1289/ehp.8527

**Published:** 2006-02-16

**Authors:** Dana B. Barr, Kent Thomas, Brian Curwin, Doug Landsittel, James Raymer, Chensheng Lu, K.C. Donnelly, John Acquavella

**Affiliations:** 1 National Center for Environmental Health, Centers for Disease Control and Prevention, Atlanta, Georgia, USA; 2 National Exposure Research Laboratory, U.S. Environmental Protection Agency, Research Triangle Park, North Carolina, USA; 3 National Institute for Occupational Safety and Health, Centers for Disease Control and Prevention, Cincinnati, Ohio, USA; 4 Department of Mathematics and Computer Science, Duquesne University, Pittsburgh, Pennsylvania, USA; 5 RTI International, Research Triangle Park, North Carolina, USA; 6 Rollins School of Public Health, Emory University, Atlanta, Georgia, USA; 7 Department of Environmental and Occupational Health, Texas A&M University System Health Science Center, College Station, Texas, USA; 8 Monsanto Company, St. Louis, Missouri, USA (retired)

**Keywords:** biomonitoring, blood, farmworker, urine

## Abstract

Although biomonitoring has been used in many occupational and environmental health and exposure studies, we are only beginning to understand the complexities and uncertainties involved with the biomonitoring process—from study design, to sample collection, to chemical analysis—and with interpreting the resulting data. We present an overview of concepts that should be considered when using biomonitoring or biomonitoring data, assess the current status of biomonitoring, and detail potential advancements in the field that may improve our ability to both collect and interpret biomonitoring data. We discuss issues such as the appropriateness of biomonitoring for a given study, the sampling time frame, temporal variability in biological measurements to nonpersistent chemicals, and the complex issues surrounding data interpretation. In addition, we provide recommendations to improve the utility of biomonitoring in farmworker studies.

Biomonitoring of exposure to pesticides involves the measurement of a pesticide(s), its metabolite(s), or reaction product(s) in biological media such as urine, blood, or blood components and tissues (Anwar 1997; Barr et al. 1999; He 1993, 1999). Although biomonitoring has been used in many occupational and environmental health and exposure studies (Acquavella et al. 2004; Arbuckle et al. 1999; Azaroff 1999; Coronado et al. 2004; Curl et al. 2002; Rees 1996; Shealy et al. 1996; Thompson et al. 2003), we are only beginning to understand the complexities and uncertainties involved with the biomonitoring process—including chemical toxicokinetics, matrix considerations, the appropriateness of the use of biomonitoring, study design, sample collection, and chemical analysis—and with interpreting the resulting data. We present an overview of concepts that should be considered when using biomonitoring or biomonitoring data, assess the current status of biomonitoring, and detail potential advances in the field that may improve our ability to both collect and interpret biomonitoring data.

To begin, we define a few critical terms and phrases used in this article. “Internal dose” is defined as the amount of a chemical that is absorbed into the body after an exposure has occurred. Although we use the term “exposure” while discussing biomonitoring, biological measurements are not actually measuring exposure as classically defined; they are used to assess exposure by estimating the internal dose. A “biomarker” is a chemical or metabolite measured in biological matrices to assess exposure to a given chemical. “Biomarker measurements” and “biological measurements” may be used interchangeably in the text and are generally expressed on a whole-volume (e.g., micrograms per liter) or a creatinine-adjusted (micrograms per gram creatinine) basis. Given many assumptions about uptake, metabolism, steady-state excretion, and other factors, biomonitoring measurements or concentrations can be used to provide a rough estimate of an internal dose, typically expressed as micrograms per kilogram per day. “Excretion” is defined as the amount of a pesticide or metabolite eliminated from the body during a given time period; excretion should not be confused with the concentration of a chemical in a single sample. A “biomolecular adduct” is defined as a covalent chemical link between the pesticide and proteins (e.g., hemoglobin, albumin, or butyryl or acetyl cholinesterase) or DNA. The terms “toxicokinetics” and “pharmacokinetics” are often used interchangeably to define the biological processing of a putative toxicant in the body; however, in this article, we use the term “toxicokinetics” to describe the behavior of a nonpersistent pesticide (NPP) in the body.

## General Toxicokinetics

After exposure to an NPP, a portion of the pesticide may be absorbed into the bloodstream, distributed among the bodily tissues, metabolized, and/or excreted. These four complex steps of absorption, distribution, metabolism, and excretion (ADME) make up the toxicokinetic process after a pesticide contacts and enters the body (Klaassen 2001). To assess human exposure to a given pesticide, measurements of the pesticide can be made after the absorption step or during each subsequent step of ADME. Biomonitoring is a measurement of the concentration or dose of the chemical during or after ADME, and its concentration level depends on the amount of the chemical that has been absorbed into the body, the toxicokinetics (ADME) of the chemical in that body, and the exposure scenario (including the time sequence of exposure and time since last exposure) (Barr et al. 2005a). Biomonitoring data are typically independent of the pathway of exposure. They integrate exposures from different routes and reflect the amount of the chemical that is in the matrix sampled, which is some portion of what actually entered the body. However, how and where a pesticide is metabolized can affect the chemical measured and the timing the sampling should occur. With knowledge of the toxicokinetics, the internal dose can then be estimated by measuring the level of a chemical, its metabolite, or its reaction product (a chemical adduct) in a biological medium. The biomarker concentration is dependent upon the matrix and when in the ADME process the biological sample is taken, but generally the sample is taken during or after the distribution step. Ideally, to link the dose with any health outcomes, we would prefer to measure the biologically effective dose, the dose at the target site that induces an effect. Often, however, the target organ is not known, and even if known, it is usually not available for sampling. In these situations, we measure the level of the chemical in another biological sample, such as urine or blood, to gauge the internal dose.

## Matrices for Biomonitoring Measurements

**Figure 1:**
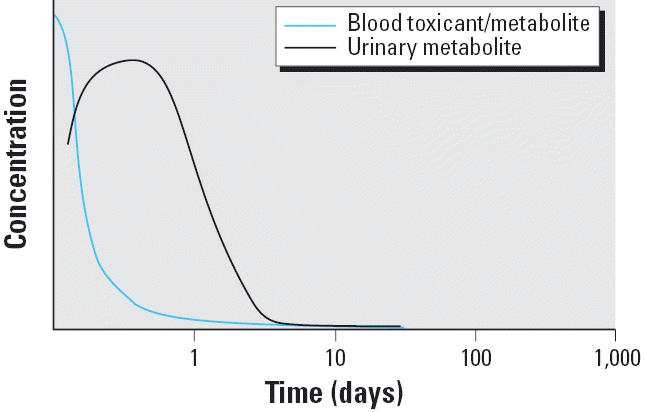
Hypothetical postexposure fate of an NPP after a single exposure, represented by the *y*-axis. Adapted from Needham and Sexton (2000).

**Figure 2:**
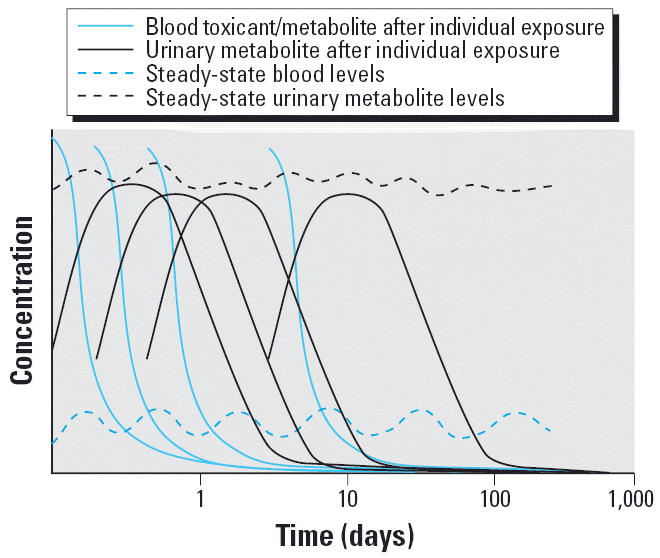
Hypothetical postexposure fate of an NPP in blood and urine after repeated (chronic) exposures.

Nonpersistent organic chemicals such as current-use pesticides can be challenging to measure from both a sample collection and a chemical analysis point of view (Barr et al. 1999, 2003, 2005a; Koch et al. 2002). The route of exposure for farmworkers, depending on the scenario, is primarily dermal; however, other routes may dominate for nonoccupational exposures. NPP may be absorbed and distributed throughout the body based on their chemical and physical properties. They are generally metabolized rapidly, and their metabolites are eliminated in urine (Figure 1). Urinary metabolites can usually be measured up to several days after an exposure has occurred. These measurements represent only a snapshot in time; thus, only exposures that occurred during the previous few days would be captured, depending upon the magnitude of the exposure. When chronic exposure occurs, the exposure is continually replenishing the chemical in the blood. Urinary elimination may reach a steady state (Figure 2); that is, the chemical or metabolite present in the urine stays at a relatively constant level. In these instances, a single urinary measurement may reflect the average exposure over a longer period. The deposition matrices, such as adipose tissue, are minor matrices for monitoring NPPs because only small amounts, if any, of the pesticides are retained in body tissues.

The two primary matrices used to assess human exposure to pesticides are urine and blood (e.g., serum, plasma, blood cells) (Angerer and Gundel 1996; Needham and Sexton 2000). The volume of blood in a person’s body is relatively constant and consistent from person to person; adults typically have about 5 L of blood (Klaassen 2001). Urine volume, of course, is not constant. One of the major advantages of using urine in biomonitoring is the ease of its collection for spot or grab (untimed) urine samples; however, collection of 24-hr urine voids can be very cumbersome and may result in non-adherence. Therefore, spot urine samples, whether first-morning voids or “convenience” samplings, are most generally used for biomonitoring purposes. The major disadvantages of spot urine samples include the variability of the volume of urine and the concentrations of endogenous and exogenous chemicals from void to void (Barr et al. 1999). How best to adjust the urinary concentrations of pesticides is the subject of continued research and much debate.

Blood has also been used as a matrix for biomonitoring (Driskell et al. 1991; Saito et al. 1984; Sancho et al. 2000). Blood measurements are usually more specific for a pesticide because the parent chemical is generally measured, as opposed to its metabolite; however, urinary measurements are equally specific if the parent compound is excreted in urine [e.g., 2,4-dichlorophenoxyacetic acid (2,4-D), glyphosate, sulfonyl ureas]. For example, if someone is exposed to chlorpyrifos, we can measure chlorpyrifos in the blood rather than its metabolite, 3,5,6-trichloro-2-pyridinol (TCPY), which is formed from more than one parent chemical (i.e., chlorpyrifos and chlorpyrifos methyl) and is also the same chemical as environmentally degraded chlorpyrifos. The blood measurement of chlorpyrifos would offer more chemical specificity than measurement of urinary TCPY. Because we know how much blood is in the body, we can calculate the body burden (i.e., the amount of chemical relative to the amount of blood in the body) more accurately than if we measure the chemical or its metabolite in urine. However, NPPs tend to have very short half-lives in blood, and the concentrations are usually about 3 orders of magnitude lower than urinary metabolite levels (Barr et al. 2002). Thus, if blood is used as a matrix, the sensitivity of the analytical method and the matrix volume available for analysis may become important. Blood can also be a valuable matrix for measuring biomolecular adducts such as DNA, hemoglobin, or albumin adducts (Anwar 1997). Typically when blood is used for this type of measurement, special sample collection and/or preparation procedures, such as washing red blood cells (hemoglobin adducts) or isolation of white cells (DNA adducts), may be required. However, if analytical requirements such as cost and speed of analysis are not critical issues, adduct measurements may provide more relevant information for relating to selected health end points such as cancer. Furthermore, adducts provide a longer window for capturing an exposure because the lifetime of an adduct in the body is largely dependent upon the lifetime of the biomolecule itself. For example, hemoglobin has a lifetime of about 120 days; thus, a hemoglobin adduct could be measured weeks or months after an exposure has occurred.

If blood testing is used, the stability of both the matrix and the target pesticide in the blood should be considered. If testing is not performed soon after sample collection, which is often the case, long-term storage of blood may be problematic, depending upon what form of blood is stored. Serum stores well at −70°C because it is low in protein and stays homogeneous. Plasma contains more proteins, which precipitate making plasma less homogeneous than serum. Whole blood does not store well because the cells tend to hemolyze. Also, many pesticides are reactive in blood; thus, the stability of the pesticide in this matrix under the desired storage conditions should be evaluated.

Saliva has also been explored as a matrix for measuring selected nonpersistent chemicals, such as atrazine and diazinon (Borzelleca and Skalsky 1980; Denovan et al. 2000; Lu et al. 1997a, 1997b, 1998, 2003). Several animal studies (Lu et al. 1997a, 1997b, 1998, 2003) have shown that pesticides can be measured in rat saliva. In these studies, atrazine, a member of triazine herbicide family, and diazinon, an organophosphorus pesticide, were measured in both saliva and plasma after controlled dose administration. The concentrations of these two pesticides in saliva and plasma were significantly correlated, and this correspondence was not affected by factors such as salivary flow rates. Denovan et al. (2000) developed a saliva sampling protocol in which multiple daily saliva samples were collected from a cohort of herbicide applicators who sprayed atrazine and other triazine herbicides in the Ohio Valley. The results indicated that saliva can be used to assess atrazine exposure and its elimination in humans. Several field studies are being conducted to evaluate saliva biomonitoring in humans. The existing data indicate that saliva levels can be considerably lower than blood levels of a pesticide, depending upon the degree of protein binding that may occur; thus, a very sensitive analytical technique is required. Further research on additional chemicals and the relation of these measurements to more commonly used approaches is required before this can be routinely used for analysis. However, measurement of pesticides in saliva has great potential because of the convenience of sampling and analysis and the potential accuracy of salivary concentrations as an indicator of tissue availability (Arcury et al. 2003; Quandt et al. 2001). These advantages would allow researchers to routinely collect multiple saliva samples in 1 day for a period of time from a larger group of individuals, allowing better characterization of within- and between-worker variability, thereby reducing uncertainties in estimating daily exposure and absorbed dose. However, issues surrounding inter- and intraindividual variability in saliva flow rates, external contamination of samples, and small sample volumes must be addressed before saliva becomes a more viable matrix for farmworker biomonitoring.

## Dermal Dosimetry versus Biomonitoring

Two exposure measurement approaches—dosimetry and biomonitoring—have generally been used in farmworker exposure assessments. Each approach has advantages and disadvantages based on information provided, uncertainty, participant burden, and resource requirements. The first approach, which is described in more detail in the environmental workgroup report (Hoppin et al. 2006), typically uses a set of dermal dosimeter measurements for an individual collected during a specific set of work activities. Collection of dermal dosimeter and other environmental samples have the advantage of providing information about specific routes of exposure and also provide exposure information that is directly related to the particular activity being monitored (Hoppin et al. 2006). However, these types of samples can measure only the potential exposure. A number of assumptions and empirical parameters about the transport of the target chemical onto and through the skin and lungs are required in order to estimate an internal dose. Unfortunately, those assumptions and empirical parameters usually dictate the magnitude of the estimated internal dose.

Biological measurements certainly can provide better evidence of the occurrence of exposure and the subsequent absorption that brings us closer to the more toxicologically relevant measure of internal dose. Nevertheless, biomonitoring is subject to toxicokinetic and exposure scenario uncertainties that may limit their use for dose estimation, assessment of exposure factors, and identification of routes of exposure. The biomarker accounts for all exposures and routes of exposure and can potentially provide information for dose estimation. A single measurement of urinary concentration can provide an indication of the presence or absence of exposure, but the quantitative calculation of absorbed dose is weakened by intraindividual genetic variability and the unknown excretion kinetics for individual pesticides under different routes of exposures (oral vs. dermal vs. inhalation).

Collection of blood or multiday urine sample collections can be burdensome; however, these measurements typically help provide a more meaningful picture of the exposure. Other pesticide exposures not related to the farmwork activities being studied may make interpretation of exposure prediction factors difficult. Interindividual differences in toxicokinetics present challenges in comparing results of metabolite measurements among different people.

## When Is Biomonitoring Appropriate?

Biomonitoring has been successfully used in many farmworker exposure studies. Studies are often focused on task-based assessments, for example, exposures resulting from uses of different chemical handling and application methods (Coronado et al. 2004; Grover et al. 1986a, 1986b), harvesting or thinning operations (Coronado et al. 2004; McCurdy et al. 1994), or uses of personal protective equipment (Ojanen et al. 1992). Other studies have examined multiple exposure factors (Acquavella et al. 2004; Arbuckle et al. 2002; Arcury et al. 2005; Hines and Deddens 2001) or relationships between dermal or environmental and biomarker measurements (Curl et al. 2002; Franklin et al. 1981; Honeycutt et al. 2001; Shealy et al. 1997). Although a number of studies have measured biomarkers of effect (Anwar 1997; Drevenkar et al. 1991; Hussain et al. 1990), fewer biomonitoring studies have been designed to assess exposures and health outcomes over either short or long time periods. Considerable knowledge of farming operations, exposure assessment, toxicokinetics, and analytical chemistry is needed for successful design, implementation, and interpretation of farmworker biomonitoring studies.

Research publications and guidelines have described considerations, procedures, and issues in farmworker biomonitoring (Fenske 1997; Nigg and Stamper 1989). Honeycutt (1986) described four criteria that must be met for deriving exposure from measuring pesticides or their metabolites in body fluids: *a*) the pesticide must be absorbed to an appropriate extent, and dermal absorption studies should be performed on appropriate species; *b*) the toxicokinetics and excretion kinetics in appropriate animals or humans must be understood; *c*) the analytical method must measure excreted parent compound or metabolites and the method must have sufficient sensitivity to detect concentrations that occur at the toxicologic no-effect level; and *d*) collection methods should be convenient for workers to gain their cooperation.

**Figure 3:**
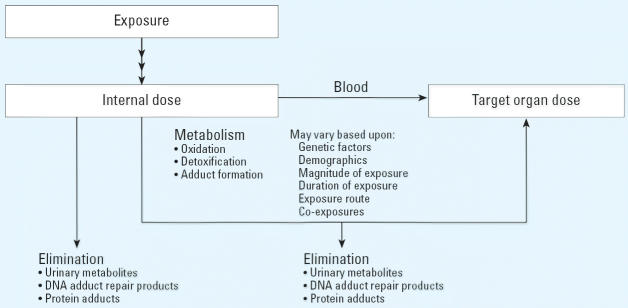
Pathway for biological measurements.

As with the criteria recommended by Honeycutt (1986), the U.S. Environmental Protection Agency (U.S. EPA 1996a, 1996b) has developed occupational and exposure test guidelines that include guidance for biological monitoring studies. The feasibility of implementing biological monitoring studies is determined by information available on the ADME of the chemical of interest. The pathway for measurements of biomonitoring of exposure in farmworker studies is shown in Figure 3.

## Designing Biomonitoring Studies

Determining whether a biomonitoring study is feasible is only the first step in designing studies that assess exposure or differences in exposure for an individual or between individuals. Successful studies require using knowledge of the ADME process and analytical chemistry to develop an implementation approach that will meet study objectives for a specific chemical. In particular, the timing of biological sample collection is very critical and highly dependent on both the exposure scenario and the uptake and elimination kinetics of the individual pesticides that may depend largely on the pesticides chemical and physical characteristics because, as stated previously, many pesticides are rapidly absorbed and eliminated with biological half-lives of 6–48 hr. Biomonitoring to assess well-defined handling or task-related exposures requires access to potential farmworker participants and sample collection shortly before and for a relatively short time (typically 1–5 days) after the monitored activity.

Assessing long-term exposures to pesticides may be of interest in studies of health effect or health outcomes. Such an assessment may be difficult to implement, particularly for NPPs with short biological half-lives. In such cases, it will be necessary to collect biological samples at multiple time points as pesticide-related activities are repeated through a season. Such studies have been reported for forestry workers (Lavy et al. 1993) and in a multiday study of exposure of farmers, their spouses, and their children to three pesticides (Acquavella J, personal communication).

Human toxicokinetic information has been reported for some chemicals, for example, 2,4-D (Sauerhoff et al. 1977), chlorpyrifos (Nolan et al. 1984), and atrazine (Buchholz et al. 1999); however, these data are lacking for most pesticides. Hence, most pesticides will require using animal data, where available, to estimate human parameters and the uncertainty of extrapolating these data to humans must be recognized. However, a properly designed biomonitoring study may provide insights into whether animal and human toxicokinetics for a given pesticide are similar. In some cases, it may be feasible to measure the parent compound in urine, blood, or saliva, but most often metabolites of parent compounds are measured in biological media. After appropriate study design and protocol development, the institutional review boards of participating groups must obtain human subjects’ approvals to ensure that the protocol complies with all national and international guidelines for the protection of research subjects.

## Sampling Time Frame

The sampling time frame for NPPs is not straightforward. Because these chemicals have short biological half-lives, the samples, whether blood, urine, or saliva, must be collected soon after the exposure in order to appropriately assess the exposure. In general, sample collection for NPP measurements should reflect the residence time of the chemical in each individual matrix. The half-lives of NPPs in blood are typically much less than in urine samples. Thus, blood samples may need to be collected within minutes or hours after the exposure, whereas urine samples may be collected several hours or, in some instances, days after the exposure. However, understanding the timing of exposure may complicate the sample collection scheme because very often the exposure time may not be easily pinpointed. For example, farmworkers may be unaware that a pesticide was applied to a particular crop or what pesticide was applied and thus would be unaware of the exposure. Further, the contact with pesticides could occur throughout the workday, intermittently during the workday, or even during one short segment of the workday. Without having information on the precise timing of exposure, the magnitude of the exposure will be difficult to estimate.

## Temporal Variability in Biological Samples

The variability of NPP levels in samples collected from an individual over time is of concern whether the sample is biological or environmental. Temporal variability can include the variation of a given chemical in multiple samples collected on a single day or can include variation among days, months, or seasons. For chronic exposures to pesticides, a single sample will likely represent a day’s exposure to a given chemical or exposure over a longer period of time, because the exposure is repeated. However, for episodic exposures, a single sample may or may not represent a single-day or longer-term exposure to a given chemical. To further complicate the process, the representativeness of a single spot sample will likely vary from chemical to chemical and among persons. In general, occupational exposures such as those encountered by farmworkers tend to be episodic, whereas environmental exposures tend to be chronic with occasional mini-episodic exposures. Farmworkers are likely to encounter both occupational and environmental exposures.

For urine matrix, a 24-hr urine sample is preferred rather than a single spot sample on a given day. Further, estimated total excretion of certain pesticides/metabolites has been shown to correlate highly with their levels in 24-hr urine samples (Acquavella J, unpublished observations). Collection of a 24-hr sample before the monitored activity and collection of 24-hr samples from 1 to 5 days after the activity have been recommended (Honeycutt 1986; U.S. EPA 1996a, 1996b). Collection and analysis of spot urine samples are sometimes used to reduce participant burden and to avoid potential confounding from additional chemical uses. If spot samples are collected, a first morning void is often preferred because the urine is more concentrated, the collection represents a longer window of accumulation (usually > 8 hr), and it is often correlated with total excretion over 24 hr (Kissel et al. 2005; Scher et al., in press). To evaluate daily, monthly, or seasonal variations of analytes in urine, sequential samples are often taken days and weeks apart to evaluate how the intraindividual variation over time compares with the interindividual variation and whether an accurate classification of exposure is possible. Temporal variation studies are important in interpreting the biomonitoring data and should be considered, at some level, in all biomonitoring studies. These data will help to determine whether multiple samples should be taken and at what intervals. In most instances, sampling for nonpersistent chemicals, whether environmental or biological, will require multiple samples taken over the course of the study at regular intervals (e.g., weekly, monthly, semiannually, etc.).

## Issues in Data Interpretation

The toxicokinetic process is complex and dynamic and may vary based on demographic variables such as age, sex, or race/ethnicity or may change with diet, coexposures (e.g., environmental chemicals, alcohol, tobacco, and medications), and certain medical conditions. The variability in the toxicokinetic process makes interpretation of biomonitoring data inherently complex. It is difficult to gauge the differences in the magnitude of exposures among individuals based upon biological measurements alone because their metabolism may play a key factor in these differences. Measurement of urinary biomarkers allows direct assessment of exposure and dose for some pesticides. For example, 2,4-D is largely unmetabolized, and > 95% is eliminated in urine (Sauerhoff et al. 1977). Multiple elimination routes and variable metabolism can complicate the measurement and interpretation of biomarkers for other chemicals. For example, atrazine has been shown to have multiple urinary metabolites (Buchholz et al. 1999). Large interindividual differences in the formation of the metabolites, and changes of the metabolite profile at different times after exposure further complicate interpretation of exposure and dose using urinary atrazine biomarkers (Buchholz et al. 1999). Other pesticides may undergo significant elimination through feces and sweat that might not be accounted for in urine collections. Understanding intra- and interpersonal variability in metabolism (e.g., the ability to appropriately oxidize molecules using cytochrome P450 enzymes or detoxify activated molecules with paraoxonase activity) and excretion will allow better assessment of the uncertainty in concentration measurements and dose estimations. Regardless, the interpretation of biological data remains a complex process and should be made with caution.

Interpretation of biological measurements can be confounded in several ways. The farmworker may have been exposed to the pesticide of interest in the days before the monitored activity. In this case, the biomarker level may not be at a baseline before sample collection in the study. In other cases, the farmworker may be exposed to the chemical in days subsequent to the monitored activity, potentially interfering with results from multiday postapplication sample collections. Correction of pre- or postactivity concentrations may be difficult or impossible unless the other exposure scenarios are well defined and the uptake and elimination kinetics well understood. Where feasible, studies of exposure resulting from specific pesticide handling or work tasks should be performed with no other handling or work task within several days before or after the monitored activity. If that is not feasible, information about the time, duration, and amount of handling and work tasks should be collected through questionnaires to allow better interpretation of the monitored activity.

Interpretation of biological measurements can also be complicated if the metabolite of interest can be formed by different parent pesticides. For example, 1-naphthol is a metabolite of both naphthalene (used as a moth repellent and also a polycyclic aromatic hydrocarbon from combustion processes) and carbaryl (Shealy et al. 1997). In this case, it may be important to obtain information regarding potential exposures to the other chemicals. The most specific biomarker available for a particular chemical should be used whenever possible to simplify interpretation. Emerging work also suggests that people can be exposed to the metabolite of a chemical in the environment (Morgan et al. 2005; Wilson et al. 2003) or through the diet (Lu et al. 2005). If the metabolite is absorbed in the body, then distinguishing between exposure to the parent chemical and the metabolite may create uncertainty in the interpretation of the measurement. Measurements to assess potential exposures to metabolites should be performed to determine whether the metabolite is present in the worker’s environment in sufficient quantities to interfere with the biomonitoring study.

Successful interpretation of biological measurements often requires collecting important information from the farmworker regarding the activities resulting in pesticide exposures. Information about the start and completion time for important activities is needed in order to place the exposure in correct relation to the biological sample collection timing. Information about the work task can be used to assess differences between farmworker measurement results. Such information might include use of specific equipment or procedures and the number of times activities were repeated. Information about other potential exposure factors may be collected to aid interpretation of results. Such factors might include use of personal protective equipment or engineering controls, hygiene activities and timing, food consumption or smoking during the work period, and possible spills or equipment leaks during pesticide handling (Quandt et al. 2006).

There are several issues regarding interpretation of urinary measurement results. For spot samples, the concentration measurement may not be representative of elimination over longer time periods because of short-term volume and excretion rate differences. For 24-hr samples, there can be a high degree of intra- and interindividual variability in 24-hr urine volume, making uncorrected comparisons of concentrations across days and between people difficult to interpret. Researchers often fail to consider that the 24-hr collection period does not necessarily translate directly to a 24-hr excretion period—a period that may be several hours shorter or longer depending on void times. Studies relying on single 24-hr samples should take the excretion period into account when comparing results among applicators.

Urinary creatinine is often used to adjust for urine volume in biomonitoring for exogenous chemical exposures. For some chemicals, such corrections have been shown to reduce uncertainties (Barber and Wallis 1986). However, for some biomonitoring studies the creatinine corrections have failed to improve, or have actually increased, uncertainties (Berlin et al. 1985). There are two reasons why creatinine corrections may not be appropriate for pesticide biomonitoring. First, there is a wide range of normal creatinine excretion in healthy adults, making it difficult to compare creatinine-adjusted results between people (Alessio et al. 1985; Barr et al. 2005b). Second, relatively large intraindividual daily variability in creatinine excretion has been reported, suggesting that even creatinine corrections between 24-hr periods for the same individual may not be appropriate (Greenblat et al. 1976). The underlying physiologic process of creatinine formation and excretion can be dependent on several factors, including age, gender, diet, exercise, muscle mass, and underlying disease (Boeniger et al. 1993). These processes may not be the same as those governing metabolism and excretion of pesticides.

Other parameters such as specific gravity or osmolality have been suggested as a way to adjust for variable urinary volumes. Another approach using excretion rates has not been widely reported but may deserve consideration and testing. Under this approach, the excretion rate is calculated for the urine sample and used in analysis rather than the concentration. To calculate the excretion rate information about the total urine void volume, the start and end times for collection of the urine sample, and the time of the previous void before starting to collect the urine sample must be known. This approach has the potential advantages of accounting for all of the excretion during a given time period and eliminating the urine volume issue. This approach might not be appropriate if pesticide or pesticide metabolite excretion is found to be dependent on internal urine production rates.

## Use of Biomonitoring in Paraoccupational Exposure Assessments

Paraoccupational exposures, often called take-home exposures, are environmental exposures that occur from the transfer of pesticides from a person who is occupationally exposed to a nonoccupational environment. Similar biomonitoring issues should be addressed when evaluating paraoccupational exposures, although the exposures may be expected to be lower in magnitude and more difficult to interpret. Urinary dialkylphosphate levels have been used in many studies, which indicate children are at risk of exposures to organophosphorus pesticides based on their parental occupation or their household proximity to farmland (Koch et al. 2002; Lu et al. 2000), self-reported residential use of pesticides by the parents (Aprea et al. 2000; Lu et al. 2001) or other factors (Aprea et al. 1996). However, the uncertainties in these measurements should also be considered to determine whether the biomonitoring measurements reflected true exposures or exposures to environmental breakdown products.

## What Can Biomonitoring Measurements Really Tell Us?

Obviously, to yield the maximum information from the biomonitoring component of a farmworker study, the ideal assessment should include multiple, longitudinal 24-hr samples after a known exposure event to a known chemical with other potentially variable factors narrowly controlled. In many farmworker studies, however, many of these variables cannot be feasibly controlled or even understood, and the costs and participant burden may be too large to bear. For example, often farmworkers do not know if any pesticide has been used on a field in which they are working. Thus, it would be nearly impossible to determine the timing of exposure. In general, a single spot-sample biomonitoring measurement can allow a cross-sectional evaluation of whether or not an exposure has occurred and some information on the magnitude of exposure, assuming the chemical measured is specific for a given exposure. To go beyond this interpretation, additional information is necessary. For example, if the exposure timing is unknown, certain activities could be recorded and the biomonitoring measurements evaluated in relation to the specific activities. Additionally, environmental or personal measurements could be taken to evaluate the potential for exposure from various activities. Multiple measurements over time may allow evaluation of the consistency of exposure, especially if the same activity is performed over that period of time.

If the biomonitoring measurements are to be used to extrapolate back to the exposure, additional information for that chemical such as toxicokinetic data, rate of intake, and rate of uptake may be required. Again, this still would provide only a single measurement in time. Multiple measurements over the duration of exposure-related activities must be taken to determine peak exposures. To relate the biomonitoring measurement to a health outcome, other required information may include population susceptibility factors, plausible mechanism of toxicity, and information on whether the exposure evaluated preceded the development of the health effect in question. Of course, such exposure estimates and health effects associations can be made without the necessary information, but the strength of the interpretation is weakened because of the additional uncertainties associated with the assumptions made in lieu of the required information.

## Specific Recommendations

To allow better harmonization of data from existing and future studies involving biomonitoring, we recommend several specific guidelines for study design and implementation and for reporting data and study findings in the peer-reviewed literature:

- The appropriateness of biomonitoring measurements in the proposed study should be considered.
- The frequency and timing of sample collection should be carefully evaluated, and an adequate number of samples to describe the exposure should be taken.
- Ideally, pre- and postexposure samples should be collected. If the timing of exposure is unknown, specific task-related activities should be recorded for each study participant.
- Ideally, 24-hr samples should be collected. If not possible, a first morning void, noting the time since last urination and the total urine void volume, should be collected. If collecting a spot sample is the only practical option, the ability of a single spot sample to predict a 24-hr value should be evaluated in a pilot study.
- Paraoccupational exposures should be evaluated alongside farmworker exposure studies.
- The most selective analytes for assessing exposures should be measured in biological samples. Measurement of less selective metabolites may allow for “class” exposure assessment that may help to more narrowly focus measures for subsequent studies.
- The most selective measurement techniques for providing robust data should be employed. However, the cost of the analysis should be carefully weighed against the sample number requirements to ensure that the overall cost is in keeping with the study budget. Fewer “selective” measurements may provide less meaningful data than a larger number of samples with more general measurements, depending upon the exposure assessment question. The reverse can also be true, depending upon the question.
- Analytic methods should be further developed allowing the analysis of potentially useful biomolecular adducts that might extend the window for capturing exposures or allow more interpretable measurements of internal doses.
- The uncertainties of biological measurements should be considered when interpreting the resulting data. The uncertainties associated with the measurements and with the interpretations and any assumptions made during the process should be explicitly stated.
